# Value of Cardiac Biomarkers in the Early Diagnosis of Takotsubo Syndrome

**DOI:** 10.3390/jcm9092985

**Published:** 2020-09-15

**Authors:** Charlotte Dagrenat, Jean Jacques Von Hunolstein, Kensuke Matsushita, Lucie Thebaud, Stéphane Greciano, Nicolas Tuzin, Nicolas Meyer, Annie Trinh, Laurence Jesel, Patrick Ohlmann, Olivier Morel

**Affiliations:** 1 Université de Strasbourg, Pôle d’Activité Médico-Chirurgicale Cardio-Vasculaire, Nouvel Hôpital Civil, Centre Hospitalier Universitaire, 67091 Strasbourg, France; jeanjacques.vonhunolstein@chru-strasbourg.fr (J.J.V.H.); matsuken_22@yahoo.co.jp (K.M.); lucie.lachmetthebaud@chru-strasbourg.fr (L.T.); Annie.trinh@chru-strasbourg.fr (A.T.); laurence.jesel-morel@chru-strasbourg.fr (L.J.); patrick.ohlmann@chru-strasbourg.fr (P.O.); 2 Department of Cardiology, Centre Hospitalier de Colmar, 68000 Colmar, France; stephane.greciano@ch-colmar.fr; 3 Department of Biostatistics, University of Strasbourg, 67091 Strasbourg, France; nicolas.tuzin@chru-strasbourg.fr (N.T.); nicolas.meyer@chru-strasbourg.fr (N.M.); 4 UMR INSERM 1230 Regenerative Nanomedicine, University of Strasbourg, 67000 Strasbourg, France

**Keywords:** takotsubo syndrome, acute coronary syndrome, myocardial infarction, B-type natriuretic peptide, troponin I, cardiac biomarkers, diagnostic score

## Abstract

Background: Bedside diagnosis between Takotsubo syndrome (TTS) and ST elevation (STEMI) and non-ST elevation (NSTEMI) myocardial infarction remains challenging. We sought to determine a cardiac biomarker profile to enable their early distinction. Methods: 1100 patients (TTS *n* = 314, STEMI *n* = 452, NSTEMI *n* = 334) were enrolled in two centers. Baseline clinical and biological characteristics were compared between groups. Results: At admission, cut-off values of BNP (B-type natriuretic peptide)/TnI (Troponin I) ratio of 54 and 329 distinguished respectively STEMI from NSTEMI, and NSTEMI from TTS. Best differentiation was obtained by the use of BNP/TnI ratio at peak (cut-of values of 6 and 115 discriminated respectively STEMI from NSTEMI, and NSTEMI from TTS). We developed a score including five parameters (age, gender, history of psychiatric disorders, LVEF, and BNP/TnI ratio at admission) enabling good distinction between TTS and STEMI (77% specificity and 92% sensitivity, AUC 0.93). For the distinction between TTS and NSTEMI, a four variables score (gender, history of psychiatric disorders, LVEF, and BNP at admission) achieved a good diagnostic performance (89% sensitivity, 85% specificity, AUC 0.94). Conclusion: A distinctive cardiac biomarker profile enables at an early stage a differentiation between TTS and ACS. A four (NSTEMI) or five variables score (STEMI) permitted a better discrimination.

## 1. Introduction

Takotsubo syndrome (TTS) can be broadly defined as a transient left ventricular dysfunction clinically sharing many features of myocardial infarction such as acute chest pain, ECG changes and troponin rise. Various sets of criteria have been proposed over time to standardize the diagnosis of TTS. Whilst the TTS pathophysiology remains uncertain, recent advances in the field have emphasized the importance of catecholamine-induced myocardial stunning involving abnormal post beta-2-adrenoreceptor signaling, triggered by physical or emotional stress and resulting in nitric oxide synthase activation [1]. TTS is also presumably associated to an acute inflammatory disorder as suggested by the systemic inflammatory response syndrome found in a large proportion of TTS patients (45%) in a recent study of 215 patients [2].

Although initially presumed to be rare, TTS is increasingly recognized as an important differential diagnosis of acute coronary syndrome (ACS), representing 1 to 2% of these patients, and up to 8 to 10% in women [3]. Two registries of 1750 and 324 patients with TTS reported that 90% and 91% respectively were woman, with mean ages of 67 and 68 years, respectively [4,5]. Patients with TTS often present with comorbidities, two of the most commonly found being psychiatric disease and malignancy [3,4,6,7]. In the interTAK registry, the prevalence of depressive and anxiety disorders were 18.8% and 8.5% in TTS, respectively, whereas they were 7.8% and 0.9% in ACS, respectively (*p* < 0.001) [4]. A recent meta-analysis of 123,563 patients estimated that 6.7% of patients had current or previous malignancy [7]. In nearly 60% of cases, TTS mimics non-ST-segment elevation myocardial infarction (NSTEMI) presentation, whilst a ST segment elevation could be evidenced in almost 40% of cases [8]. In clinical practice, the differential diagnosis between TTS and ACS remains often difficult to assess and requires complementary examinations including renewed echocardiography, cardiac magnetic resonance imaging (MRI), or myocardial tomography. Recent cases reports have clearly underline the causality dilemma when TTS and myocardial infarction (MI) coexist and how MI especially in its aborted form may be indistinguishable from TTS [9]. Owing to the extreme frailty of TTS patients that often precludes the realization of cardiac catheterization, non-invasive criteria are needed. In TTS, the troponin (Tn) rise in serum is low, contrasting with the dramatic extent of myocardial dysfunction [10]. Conversely, plasma B-type natriuretic peptide (BNP) and NT-proBNP are substantially elevated, and their level is correlated to the degree of ventricular wall motion abnormality [11,12]. Preliminary data obtained in small cohorts have suggested that the BNP/Tn ratio on admission might be useful to discriminate TTS, STEMI, and NSTEMI [13,14,15,16].

In the present study, we sought to determine whether a distinctive cardiac biomarker profile exists during TTS enabling at an early stage, the differentiation between TTS and various myocardial infarction patterns. In addition, the diagnosis performance of various non-invasive scores based on routinely available parameters was examined to improve the distinction between TTS and ACS.

## 2. Materials and Methods

### 2.1. Study Design and Population

We conducted a retrospective, multicentric study from January 2008 to August 2017 in the Hopitaux Universitaires de Strasbourg and from January 2011 to December 2014 in the Hospices Civils de Colmar, France. Patients with suspected TTS were identified from 34,037 coronary angiograms recorded in the Cardiac Catheterization Laboratory database of the University Hospital of Strasbourg between January 2008 and August 2017. The database was queried using the key words “stress”, “takotsubo”, or “catecholamine”. The patients from the Hospices Civils de Colmar were eligible for the diagnosis of TTS after reviewing all cases of patients admitted in the intensive care unit for ACS. This review was made with the help of a local registry prospectively set up to enroll all TTS cases. The diagnosis of TTS was made according to the interTAK criteria; exclusion criteria comprised a diagnosis of acute coronary occlusion, percutaneous intervention, myocarditis, valve replacement during the hospital phase, tachycardia-induced cardiomyopathy, and cardiac arrest at first medical contact. Two cardiologists reviewed all the cases and the diagnosis of TTS was made based on a consensus agreement.

TTS patients were compared with ACS patients hospitalized in the Hopitaux Universitaires de Strasbourg during the same period of time. To ascertain that ACS patients represented type 1 myocardial infarction, the analysis of ACS patients was restricted to patients treated by percutaneous coronary intervention (PCI). All these patients fulfilled the criteria of the Joint European Society of Cardiology/American College of Cardiology/American Heart Association for the Fourth definition of myocardial infarction (2018) [17].

The study protocol was approved by the Institutional Review Board of the University.

### 2.2. Clinical Assessment

Patients’ data were collected by careful reviewing of their electronic medical records. We registered baseline characteristics, cardiovascular risk factors, medical history, medications, ECG, routine biological analyses, and data from coronary angiography and ventriculography. Multiplane coronary angiography was performed in all patients using the standard techniques. Left ventricular ejection fraction (LVEF) was assessed using ventriculography results and 2D-echocardiography according to the Simpson biplane method. Right ventricle involvement was defined by a segmental wall motion alteration of the right ventricle. Three patterns of quantitative TnI and BNP were identified: at admission, at peak, and at discharge. The ratios of BNP/TnI were computed using the first concomitantly available levels of TnI and BNP. Blood samples were analyzed using the Access 2 (Beckman) analyzer in the Colmar center and the Centaur XPT (Siemens) in the Strasbourg center.

### 2.3. Outcomes

In-hospital events were collected by reviewing electronic medical records.

After hospital discharge, follow-up was obtained for all patients using standardized telephone interviews with a cardiologist or another physician. In cases of death, the cause was ascertained by thorough review of all available clinical information at the time of death. Cardiac death was defined as any death with demonstrable cardiac cause. We recorded several follow-up variables including new hospitalization for heart failure, TTS recurrence, 30-day, and 1-year mortality from major cardiac event, from malignancy disease or from any cause. Follow up LVEF was assessed using echocardiography or MRI.

### 2.4. Statistical Analysis

Continuous variables were expressed as mean +/− standard deviation, and categorical variables as frequencies and percentages. Continuous variables between all groups were compared using ANOVA or Kruskal–Wallis test, as appropriate. Pearson’s Chi-squared test was used to compare categorical variables. Continuous variables were analyzed for normal distribution using Shapiro–Wilk test or graphically. Post hoc analyses were performed, using Tukey’s method for continuous variables and Chi-squared test with a Bonferroni correction for categorical variables. Analysis of the receiver operating characteristic (ROC) curve enabled assessment of group comparisons. Performance indicators and thresholds for test measurements corresponding to Youden index maximizations were calculated. The calculation of the volume under the ROC surface has been analyzed for comparison of three groups. Propensity score (PS) matching was also developed using age and gender. New diagnostic scores for TTS–STEMI and TTS–NSTEMI differentiation were built as follows: (i) Selection of variables using ROC curves and Logistic regression analysis to define the more accurate parameters to discriminate patients and to define equations. On a practical perspective, we decided to include a maximum of 5 variables in each score. We created two scores for each diagnosis, the first using continuous variables. For the second score, continuous variables were transformed in qualitative ones. The cut-off value was defined according to the best sensitivity and specificity on ROC curves. The second score was simplified to be friendly user in daily practice. The area under the receiver-operating characteristic curve (AUC) were compared according to the DeLong technique. Univariate and multivariate logistic regression analyses were performed to identify independent predictors of 30-day and 1-year mortality. Factors associated with a *p* value <0.05 in univariate analysis were implemented into a backward stepwise model using removal criteria of 0.10. The significance level was set at 5%. Statistical analyses were performed using the R software version 3.4.0.

## 3. Results

### 3.1. Baseline Characteristics of the Whole Cohort

**Figure 1:**
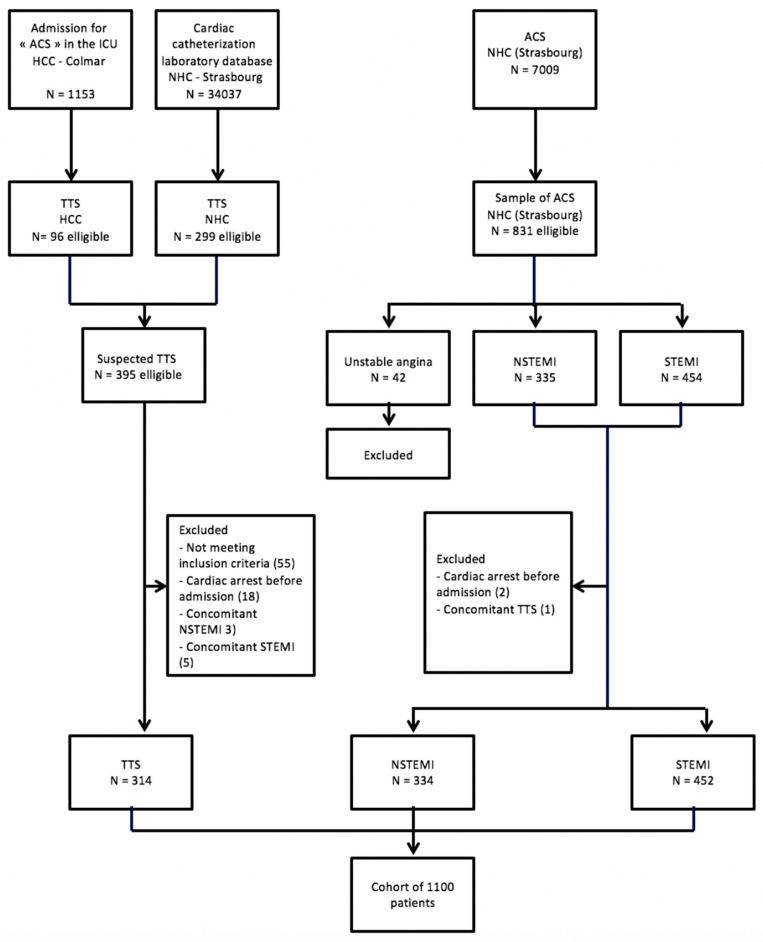
Flowchart Study. ACS, acute coronary syndrome; HCC, hospices civils de Colmar; NHC, Nouvel Hôpital Civil; NSTEMI, non ST segment elevation infarction; STEMI, ST segment elevation myocardial infarction; and TTS, Takotsubo syndrome.

There were 1100 patients enrolled in the study (flowchart: Figure 1). A total of 314 TTS patients met the inclusion and exclusion criteria and were included in the analyses. They were compared to 831 ACS patients (NSTEMI *n* = 334, STEMI *n* = 452). Their baseline demographics and clinical characteristics are reported in Table 1.

As expected, TTS affected predominantly postmenopausal women (81.8%), with a mean age of 70 years ± 13 (Table 1). By contrast, ACS patients were younger, especially in the STEMI subgroup, and mostly men. Malignancy history, psychiatric disorders and atrial fibrillation were more frequently recorded in TTS patients. A drastic impairment at admission of LVEF could be evidenced in TTS that contrasts with a mild myocardial dysfunction in ACS sub-groups. At discharge, LVEF recovery was observed in TTS patients (52 ± 12%).

Inflammatory response as assessed by CRP and leukocytes levels was enhanced in TTS patients.

### 3.2. Propensity Score Matching (Supplementary Files)

Patients were enrolled in the PS analysis after matching for age and gender. The differences in characteristics, biological patterns, and outcomes between the three subsets remained similar before and after PS matching. (Tables S1–S4).

### 3.3. Outcomes

In-hospital outcome and at one-year follow-up are given in Table 2. In hospital mortality, cardiogenic shock and supraventricular tachycardia were more frequent in the TTS sub-group. At follow-up, higher 30-day and one-year mortality could be established in TTS patients. There was no difference in cardiovascular mortality at one year. TTS recurrence was evidenced in 10 patients (3.2%). Univariate and multivariate analyses were performed to identify independent predictors of 30-day and 1-year mortality. The biomarkers (including BNP) did not predict the mortality (Tables S5 and S6).

### 3.4. Value of the BNP and the Troponin I Kinetics in the Differential Diagnosis between TTS, STEMI, and NSTEMI

**Figure 2:**
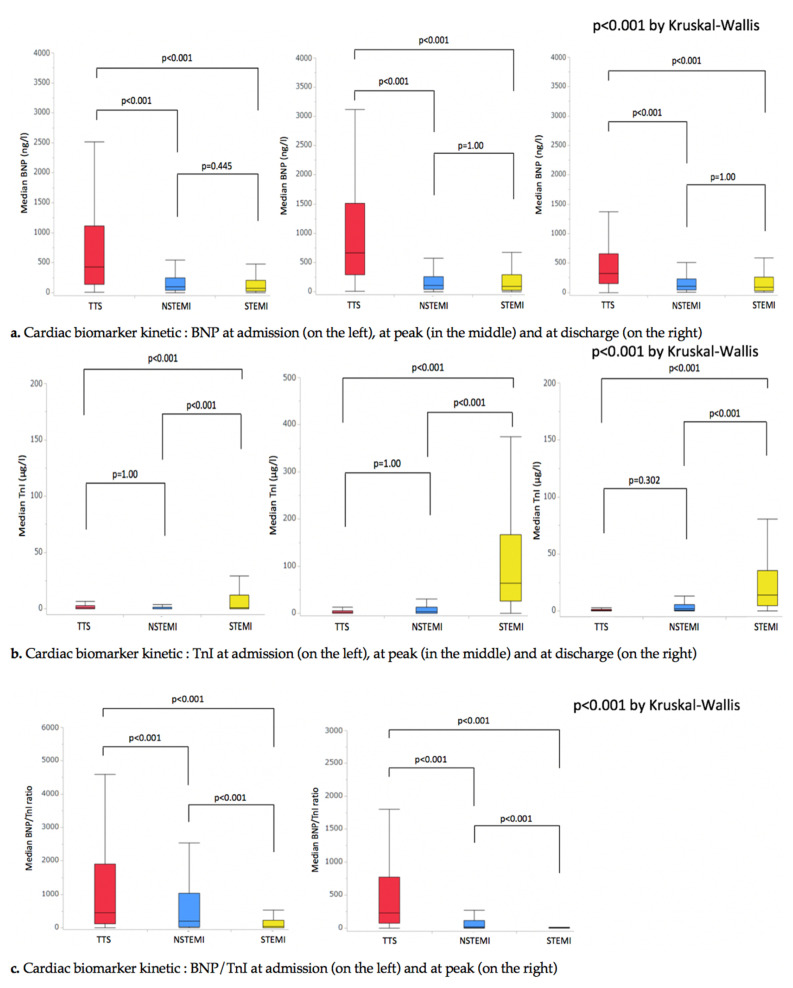
Cardiac biomarker kinetic; BNP, B-natriuretic peptide; NSTEMI, non ST segment elevation infarction; STEMI, ST segment elevation myocardial infarction; Tn, Troponin; and TTS, Takotsubo syndrome.

In TTS, higher BNP levels at admission, at peak and at discharge could be evidenced (Table 1, Figure 2a). No differences in troponin levels and kinetics between TTS and NSTEMI were revealed (Table 1, Figure 2b). Troponin levels were higher at admission, at peak, and at discharge in the STEMI subset.

### 3.5. Value of the BNP/Troponin Ratio in the Differential Diagnosis between TTS, STEMI, and NSTEMI

**Figure 3:**
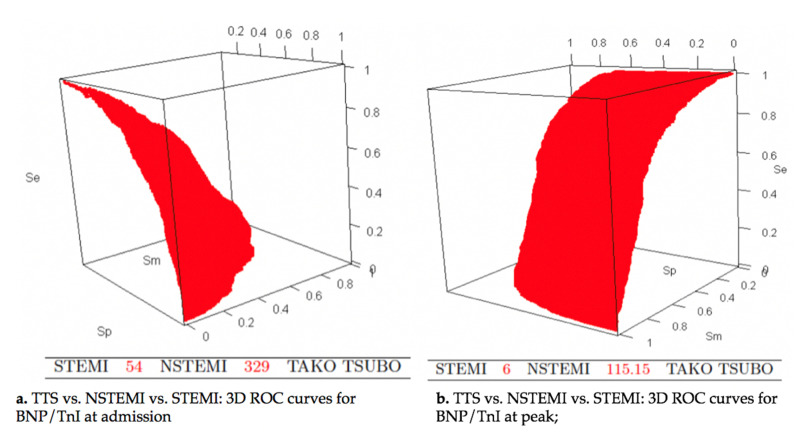
TTS vs. NSTEMI vs. STEMI: 3D receiver operating characteristic (ROC) curves for BNP/TnI at admission and at peak; BNP, B-natriuretic peptide; NSTEMI, non ST segment elevation infarction; STEMI, ST segment elevation myocardial infarction; Tn, Troponin; and TTS, Takotsubo syndrome.

At admission, cut-off values of BNP/TnI ratio of 54 and 329 distinguished respectively STEMI from NSTEMI, and NSTEMI from TTS. The probabilities of accurate classification in the STEMI subset, NSTEMI subset and TTS subset were respectively 0.616, 0.341, and 0.565. The corresponding positive predictive values were 0.649, 0.383, and 0.471, respectively (Figure 3a).

Best differentiation between TTS and ACS was obtained by the use of BNP/TnI ratio at peak (Figure 2c and Figure 3b). A cut-off value of 6 discriminated STEMI from NSTEMI, and 115 distinguished NSTEMI from TTS. The diagnostic performances were better, with the probabilities of accurate classification being 0.759 in the STEMI group, 0.547 in the NSTEMI group, and 0.684 in the TTS group. The positive predictive values were respectively 0.8359, 0.488, and 0.670.

### 3.6. Value of the BNP/Troponin Ratio in the Differential Diagnosis between TTS and STEMI

At admission, a BNP/TnI ratio greater than 68 enabled the distinction between TTS and STEMI with a sensitivity of 82% and a specificity of 63% (Table 3, Figure S1a). A better distinction between these two subsets was obtained by the use of peak levels, with a cut-off value of 39 (sensitivity of 88% and specificity of 94%) (Table 3, Figure S1b).

### 3.7. Value of the BNP/Troponin Ratio in the Differential Diagnosis between TTS and NSTEMI

A BNP/TnI ratio at admission greater than 196 excluded NSTEMI with a sensitivity of 66% and a specificity of 56% (Table 3, Figure S2a). When considering the peak value, a BNP/TnI ratio of 76 enabled the distinction between TTS and NSTEMI with a sensitivity of 79% and a specificity of 66% (Table 3, Figure S2b).

### 3.8. Diagnostic Scores for TTS and STEMI Distinction

In order to improve the differentiation between TTS and STEMI, two distinct predictive scores were built. Candidate variables for the diagnosis of TTS were examined using logistic regression analysis. In addition, these variables had to be non-invasive and commonly reported in medical files. For each selected variable, area under the curve and odds ratio (OR) were compared.

The variables with best discrimination performance were age, gender, history of psychiatric disorder, the LVEF at admission, and the BNP/TnI ratio at admission. The first score, using continuous variables enabled good diagnostic performance, with a cut-off set at 0.385 (specificity 84% sensibility 88%) (AUC 0.93 (95% CI 0.91–0.95)) (Table 3, Figure S3).

$$Score=\;\frac{\exp(-0.031+0.013\times age+0.0003\times \frac{BNP}{Tn\;I}-0.077\times LVEF+2.71\times 1\left(gender=1\right)-2.77\times 1\left(psy+=1\right))}{1+\exp(-0.031+0.013\times age+0.0003\times \frac{BNP}{Tn\;I}-0.077\times LVEF+2.71\times 1\left(gender=1\right)-2.77\times 1\left(psy+=1\right))}$$

(*gender* = 1) equal 1 if the patient is a woman, and equal 0 if the patient is a man.

(*psy*+ = 1) equal 1 if the patient presents history of psychiatric disorder, and equal 0 otherwise.

**Figure 4:**
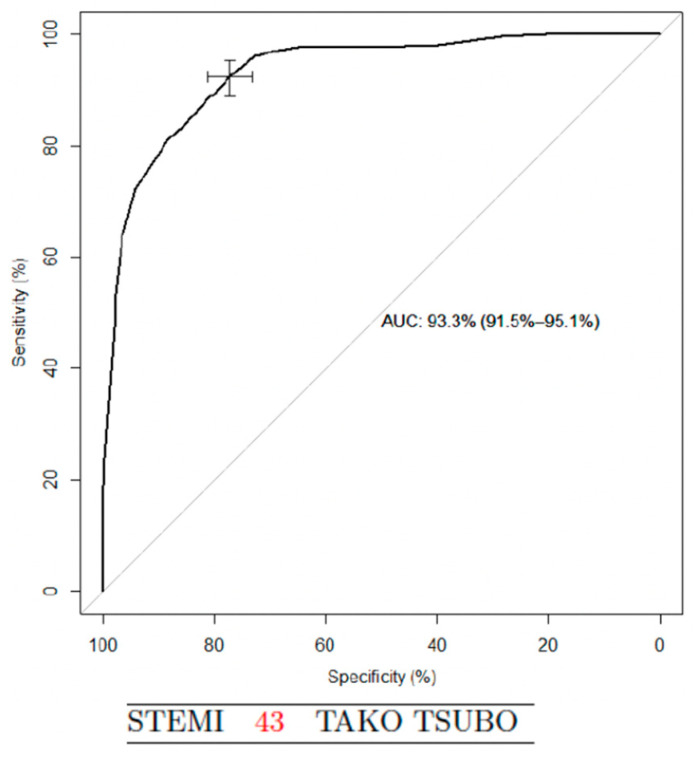
TTS vs. STEMI, ROC curve for the simplified score; STEMI, ST segment elevation myocardial infarction; and TTS, Takotsubo syndrome.

The second score, simplified, was derivate from the five variables. Points were assigned to each criterion: history of psychiatric disorder 28 points, female sex 25, BNP/TnI ratio > 67 23, LVEF < 50% at admission 19, and age > 66 years 5. Points were added in a given patient to result in a score value ranging from 0 to 100. Using a cut-off value of 43 score-points, sensitivity was 92% and specificity was 77% for the presence of TTS (AUC 0.93 (95% confidence interval 0.92–0.95)) (Figure 4 and Table 3).

The comparison of the two scores with DeLong test, showed no significant difference in AUC comparison (= 0.32).

### 3.9. Diagnostic Scores for TTS and NSTEMI Distinction

Selected variables were gender, history of psychiatric disorder, LVEF at admission and the BNP at admission. BNP at admission was integrated in the score because its AUC was better than BNP/TnI ratio AUC. Using a cut-off value of 0.441, the sensitivity was 90% and the specificity 85% (AUC 0.94, 95% CI 0.92−0.96) (Table 3, Figure S4).

$$Score=\;\frac{\exp(-0.031+0.013\times age+0.0003\times \frac{BNP}{Tn\;I}-0.077\times LVEF+2.71\times 1\left(gender=1\right)-2.77\times 1\left(psy+=1\right))}{1+\exp(-0.031+0.013\times age+0.0003\times \frac{BNP}{Tn\;I}-0.077\times LVEF+2.71\times 1\left(gender=1\right)-2.77\times 1\left(psy+=1\right))}$$

1 (*gender* = 1) equal 1 if the patient is a woman, and equal 0 if the patient is a man.

1 (*psy*+ = 1) equal 1 if the patient presents a history of psychiatric disorder, and equal 0 otherwise.

**Figure 5:**
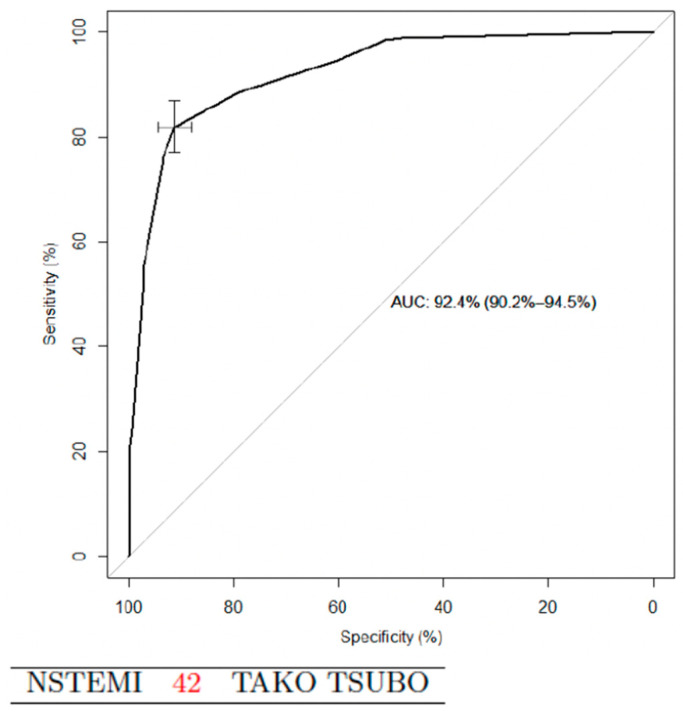
TTS vs. NSTEMI, ROC curve for the simplified score; NSTEMI, non ST segment elevation myocardial infarction; and TTS, Takotsubo syndrome.

The simplified score was established with the same four variables. Points assigned to each criterion were: history of psychiatric disorder 33, female sex 32 points, LVEF < 50% 24, and BNP at admission > 300 11 points. Using a cut-off value of 42, the sensitivity was 82% and specificity was 91% for the presence of TTS (Figure 5 and Table 3).

The comparison of the scores AUC demonstrated that the first score was superior to the simplified one (*p* = 0.01).

## 4. Discussion

The present findings drawn from a sizeable cohort of TTS patients indicate that distinctive cardiac biomarker profiles exist during TTS, STEMI, and NSTEMI (Figure 2). However, significant overlap of biomarkers value or ratio exists and precludes their use in daily practice. Simplified scores, based on commonly available non-invasive parameters on admission, allow a good discrimination between TTS and ACS.

While conceptually sounds, the monitoring of cardiac biomarkers such as troponin I or T, neuropeptides appears of limited utility in the bedside diagnosis of TTS at an early stage. TTS is characterized by a drastic elevation of neuropeptides levels that contrasts with mild elevation of cardiomyocyte necrosis markers. In addition, owing to their wide variations, the study of inflammatory markers such as CRP or leukocytes did not allow a good discrimination between TTS and ACS.

Previous studies have compared the cardiac biomarker profile of patients with TTS and ACS [13,14,15,16]. In those reports, the use of a large panel of assays (BNP or NT pro-BNP, troponin I or T, CPK) limits the possibility to compare their diagnostic performance. Other flaws are represented by the very limited size of these studies or by the exclusion of a sizeable proportion of patients admitted for suspected ACS or TTS (such as chronic kidney disease patients or patients with non-anterior STEMI.). In the study by Doyen and coworkers, TTS and STEMI were discriminated according to a BNP/TnI cut-off at peak of 159 (95% sensibility and 98% specificity) as compared to a 39 cut-off reported in the present study (88% sensibility and 94% specificity). In the work by Doyen, a ratio higher than 310 distinguished TTS from NSTEMI with 82% sensibility and 71% specificity as compared to the 76.5 cut-off described in our own experience (66% sensibility and 79% specificity). Discrepancies may rely on the use of different assays with different upper limit values, the exclusion of non-anterior myocardial infarction or patients with GFR under 30 mL/min/1.73 m^2^ in the original report by Doyen. In general, better discrimination was obtained by the study of BNP/Tn I at peak with respect to the values measured at an early stage. When considering the context of STEMI, any additional delay in the revascularization process could not be allowed and definitively precluded the use of the study of BNP/Tn ratio at peak as a meaningful tool in the decision making process. Conversely, in the setting of NSTEMI, where non-urgent revascularization generally prevailed, cardiac biomarkers kinetic appeared more useful. Nevertheless, in that context, the modest sensitivity and specificity of BNP/Tn ratio constituted an important drawback.

On a clinical point of view, characteristics of an “ideal” score comprised its easiness, the possibility to be performed at an early stage using non-invasive parameters, and excellent diagnosis performance enabling the exclusion of ACS. Ultimate finalities of such scores are to avoid unneeded cardiac catheterization but also to limit the prescription of dual antiplatelet therapy in a frail population characterized by a huge bleeding risk (Figure S5). Another potential advantages of an early diagnosis of TTS would be the avoidance of catecholamine and nitrate administration, and initiation of appropriate therapy such as beta-blockers and angiotensin-converting-enzyme inhibitors until full recovery of LVEF.

In our study, the constitution of a sizeable cohort of all-comers ACS patients or TTS allowed the identification of pertinent discriminating variables. Age, gender, history of psychiatric disorder, admission BNP/TnI ratio, and LVEF enabled the construction of a non-invasive score enabling TTS and STEMI discrimination (AUC 0.93). Moreover, when using a simplified score using continuous variables, an excellent diagnostic performance was maintained. The score enabling the distinction between NSTEMI and TTS comprised gender, history of psychiatric disorder, BNP, and LVEF at admission. In that setting, the score using continuous variables remained superior to the simplified one, with 90% sensitivity and 85%, specificity (AUC of 0.94). Importantly, all of these scores comprised widely accessible parameters enabling a daily practice use. Up to now, the InterTAK Diagnostic Score, based on the analysis of the International Takotsubo Registry remains the first attempt to provide a useful tool for TTS diagnosis, based on simple clinical parameters [18]. This score was based on a smaller population than the present one (TTS *n* = 218, ACS *n*= 436). The InterTAK scores comprised seven variables including female sex, emotional trigger, physical trigger, absence of ST-segment depression (except in aVR lead), psychiatric disorders, neurologic disorders, and QT prolongation. In the initial description, diagnosis performance of this score appeared of interest with an AUC of 0.90. A cut off value yielded a sensitivity of 89% and 91% specificity. However, as recognized by the authors, performance diagnosis appeared to be lower in daily clinical practice. With respect to the original interTAK score, the present score appears to be a simpler alternative (4 to 5 variables compared to seven) but requires the measurement of LVEF by transthoracic echocardiography and the biological assessment of TnI and BNP values at admission. Importantly, the present score was based on the study of a larger, unselected population and it enabled the distinction between TTS and STEMI, and TTS and NSTEMI (TTS *n* = 314, STEMI *n* = 452, NSTEMI *n*= 334 vs. TTS *n* = 218, ACS *n* = 436 in the InterTAK registry). Both scores underlined the importance of psychiatric disorders in the early recognition of TTS. In our cohort, psychiatric disorders could be evidenced in a large proportion of TTS patients (30.8%) but only on few ACS patients (2%).

Interestingly, we found that the rate of psychiatric and neurologic disorders was 15 times higher among TTS patients than among patients with ACS. In the interTAK registry, their prevalence was 2-fold higher [4]. This difference is probably due to the different methods in which psychiatric illnesses were diagnosed. After psychiatric consultations, we included patients with “psychiatric disease” according to DSM-5 criteria, including depressive disorders, anxiety disorders, but also addictive disorders, psychotic disorders, and dementias, not mentioned in the interTAK registry. Psychiatric disease was also more frequent among patients with cancer in comparison to patients without cancer (18% vs. 9%, *p* value < 0.001).

Another crucial issue relies on the prognosis of the TTS. Initially described as totally benign, recent reports have emphasized the adverse long-term outcome associated with TTS [19]. In the experience reported by Stiermaier and coworkers, a 12.5% one-year mortality with a significant increase of non-cardiac death was evidenced in TTS patients [20]. Likewise, in the large cohort analyzed by Templin and coll. TTS one year mortality was around 10% [4].

In our experience, one-year mortality was even higher reaching 16.3% of TTS patients. In our cohort, patients were admitted to both the cardiology unit and the critical department with a large proportion of oncologic patients [6]. Patient’s initial severity was also underlined by the high rate of initial cardiogenic shock. Altogether, these data clearly emphasized that TTS should not be consider as a benign condition. In addition to the initial myocardial dysfunction, significant comorbidities including malignancies, psychiatric disorders, and frailty are associated with TTS and could impact by themselves mid-term prognosis.

### Study Limitations

The present study shares inherent limitations of any retrospective, post hoc analysis. We based the diagnosis of TTS on interTAK criteria rather than the classic Mayo Clinic’s criteria. However, the main difference relies on the recognition that TTS may occur in a variety of illness including pheochromocytoma, ACS [9], or bleeding. In our experience the coexistence between ACS (in its aborted form) and TTS remained very rare. However, distinctive parameters between type-2 MI and TTS were not studied. ACS patients were all treated by PCI, allowing only type-1 MI inclusion. Analysis of some parameters enabling the discrimination between TTS and ACS were lacking. For instance, emotional trigger, physical trigger, and QT interval measurement were not recorded in the ACS cohort. We therefore could not validate the interTAK score in our population. Patients with a diagnosis of TTS only performed a cardiac MRI when a differential diagnosis of myocarditis or type 1 myocardial infarction was suspected. We did not differentiate the groups after matching on the initial ECG changes with and without ST segment elevation. The duration of symptoms at the time of admission was not documented in the present study and as BNP and troponin rise at different rates after onset of TTS and of MI, the validity of each of their findings can be time dependent. Moreover, serial measurements of BNP and Tn levels were not standardized. We reported assay of BNP and not NT-proBNP, as it was not available in our center at the time of the inclusion of the patients. Finally, validation of the scores was not performed in an external prospective cohort.

## 5. Conclusions

In conclusion, distinctive cardiac biomarker profile exists during TTS and enables a distinction between TTS and several ACS pattern. However, significant overlap of biomarkers values exists and precludes their use in daily practice. Simplified scores, based on commonly available non-invasive parameters (age, gender, psychiatric disorder, LVEF, and cardiac biomarker) allow a good discrimination between TTS and ACS.

## Figures and Tables

**Table 1:** Baseline characteristics of Takotsubo and ACS patients.

	TTS Patients (*n* = 314)	STEMI Patients (*n* = 452)	NSTEMI Patients (*n* = 334)	*p* Value	*p* Value STEMI vs. TTS	*p* Value NSTEMI vs. TTS	*p* Value STEMI vs. NSTEMI
**Demographics**							
Age	70.7 ± 13.4	62.1 ± 13.8	67.9 ± 13.9	<0.001	<0.001	0.024	<0.001
Female gender, *n* (%)	257 (81.8)	106 (23.4)	81 (24.2)	<0.001	<0.001	<0.001	1
**CVD risk factors**							
Hypertension, *n* (%)	180 (57.3)	229 (50.7)	234 (70.1)	<0.001	0.232	0.002	<0.001
Diabetes mellitus, *n* (%)	65 (20.7)	94 (20.8)	96 (28.7)	0.016	1	0.055	0.034
Dyslipidemia, *n* (%)	127 (40.5)	185 (40.9)	185 (55.4)	<0.001	1	<0.001	<0.001
Current tobacco use, *n* (%)	59 (18.8)	208 (46)	103 (30.8)	<0.001	<0.001	0.001	<0.001
Past tobacco use, *n* (%)	67 (21.3)	119 (26.3)	105 (31.4)	0.014	0.370	0.013	0.387
**Background**							
History of malignancy, *n* (%)	82 (26.1)	31 (6.9)	23 (6.9)	<0.001	<0.001	<0.001	1
History of psychiatric disease, *n* (%)	95 (30.4)	10 (2.2)	6 (1.8)	<0.001	<0.001	<0.001	1
History of AF, *n* (%)	50 (15.9)	22 (4.9)	39 (11.7)	<0.001	<0.001	0.413	0.019
History of stroke, *n* (%)	32 (10.2)	31 (6.9)	43 (12.9)	0.017	0.328	0.978	0.019
History of PAD or ischemic heart disease, *n* (%)	70 (22.4)	77 (17.0)	105 (31.4)	<0.001	0.228	0.031	<0.001
Chronic kidney disease, *n* (%)				<0.001	0.63	<0.001	0.04
moderate (DFG 30–60)	70 (22.3)	49 (10.8)	53 (15.9)				
severe (15–30)	11 (3.5)	12 (2.6)	9 (2.7)				
terminal (<15)	9 (2.9)	3 (0.7)	2 (0.6)				
**Hemodynamic parameters**							
Heart rate, bpm	90.2 ± 23	77.5 ± 15.6	76 ± 21	<0.001	<0.001	<0.001	1
SBP, mmHg	126.1 ± 28	128.6 ± 22	143.8 ± 28.1	<0.001	<0.001	<0.001	1
**Electrocardiogram at admission**							
Q wave, *n* (%)	163 (52.8)	96 (47.3)	6 (1.9)	<0.001	0.723	<0.001	<0.001
**Echocardiography**							
LVEF at admission, %	38.5 ± 12	50 ± 11.2	54.1 ± 11.2	<0.001	<0.001	<0.001	<0.001
RV involvement, *n* (%)	15 (5.1)	48 (10.6)	10 (3)	<0.001	0.029	0.659	<0.001
**Biology at admission**							
Tn I, ng/L	1 [2.7]	1.3 [12]	0.4 [1.5]	<0.001	<0.001	1	<0.001
BNP, ng/mL	427.5 [967]	70 [183.2]	99.5 [199]	<0.001	<0.001	<0.001	0.445
CRP, mg/L	13 [50.9]	3 [9.7]	3 [8.8]	<0.001	<0.001	<0.001	0.404
Leukocytes, G/L	12.1 ± 5.8	12 ± 4	9.8 ± 4.7	<0.001	0.886	<0.001	<0.001
Hemoglobin, g/dL	12.8 ± 2.7	14.1 ± 1.8	13.9 ± 1.8	<0.001	<0.001	<0.001	0.138
Creatinine serum μmol/L	68 [38.5]	73.8 [25]	76 [27.5]	0.437	-	-	-
GFR, mL/min/m^2^	73.9 ± 27.9	80.8 ± 17.8	77.6 ± 18.8	<0.001	<0.001	0.073	0.091
Plasma sodium level, mmol/L	137.4 ± 4.7	137.5 ± 3	138.2 ± 3.2	0.005	0.952	0.012	0.014

Data presented were number (percentage); quantitative variables as median [interquartile interval] or mean ± 95% confidence interval. AF, atrial fibrillation; CRP, C-reactive protein; CVD, cardiovascular disease; DBP, diastolic blood pressure; GFR, glomerular filtration rate; LVEF, left ventricular ejection fraction; NSTEMI, non ST segment elevation myocardial infarction; PAD, peripheral artery disease; RV, right ventricle; SBP, systolic blood pressure; STEMI, ST segment elevation myocardial infarction; and TTS, Takotsubo syndrome.

**Table 2:** In hospital outcome and follow up.

	TTS Patients (*n*= 314)	STEMI Patients (*n*= 452)	NSTEMI Patients (*n*= 334)	*p* Value	*p* Value STEMI vs. TTS	*p* Value NSTEMI vs. TTS	*p* Value STEMI vs. NSTEMI
**In-hospital events**							
Cardiogenic shock, *n* (%)	44 (14)	14 (3.1)	1 (0.3)	<0.001	<0.001	<0.001	0.009
SVT, *n* (%)	78 (24.4)	12 (2.6)	10 (3)	<0.001	<0.001	<0.001	1
Sustained VT, *n* (%)	4 (1.3)	1 (0.2)	1 (0.3)	0.115	-	-	-
VF, *n* (%)	3 (1.0)	2 (0.4)	2 (0.6)	0.110	-	-	-
Torsade de Pointe, *n* (%)	2 (0.6)	0 (0)	0 (0)	0.081	-	-	-
3-degree AV block, *n* (%)	2 (0.6)	8 (1.8)	1 (0.3)	0.092	-	-	-
In hospital mortality, *n* (%)	23 (7.3)	9 (2)	6 (1.8)	<0.001	0.001	0.003	1
Hospital length of stay, *n* (days)	8.0 [7.0]	5.0 [5.0]	4.0 [5.0]	0.511	<0.001	<0.001	1
**Follow up events**							
Hospitalization for acute HF, *n* (%)	33 (10.8)	25 (5.8)	17 (5.4)	0.019	0.337	0.042	1
30-day mortality, *n* (%)	20 (6.7)	12 (2.7)	8 (2.4)	0.005	0.027	0.033	1
1-year mortality, *n* (%)	43 (16.3)	21 (4.7)	22 (6.6)	<0.001	<0.001	<0.001	0.804
1-year CV mortality, *n* (%)	15 (5.1)	12 (2.7)	9 (2.7)	0.149	-	-	-

Data presented were number (percentage); quantitative variables as median [interquartile interval] or mean ± 95% confidence interval. AV, atrio-ventricular; CV, Cardiovascular; HF, heart failure; NSTEMI, non ST segment elevation myocardial infarction; STEMI, ST segment elevation myocardial infarction; SVT, supraventricular tachycardia; TTS, Takotsubo syndrome; VF, ventricular fibrillation; and VT, ventricular tachycardia.

**Table 3:** Cut-off values with corresponding specificity and sensitivity.

	Cut-Off Value	Se	Sp	NPV	PPV	AUC, (%)	95% Confidence Interval, (%)
**TTS vs. STEMI**							
BNP/Tn I at admission	68.25	0.8216	0.6371	0.8395	0.6071	79.9	(76.5–83.3)
Peak BNP/peak TnI	39.25	0.8810	0.9362	0.9188	0.9056	96.0	(94.6–97.3)
Diagnostic score	0.3851	0.8788	0.8386	0.9158	0.7759	92.6	(90.7–94.5)
Simplified diagnostic score	43	0.9242	0.7735	0.9413	0.7219	93.3	(91.5–95.1)
**TTS vs. NSTEMI**							
BNP/TnI at admission	196.25	0.658	0.5589	0.6434	0.5747	61.9	(57.2–66.5)
Peak BNP/peak TnI	76.5	0.7857	0.6578	0.7586	0.6916	75.8	(71.9–79.6)
Diagnostic score	0.4406	0.8951	0.8513	0.8936	0.8533	93.8	(92.0–95.7)
Simplified diagnostic score	42	0.8192	0.9138	0.8494	0.8950	92.4	(90.2–94.5)

Data presented were number or percentage (%). AUC, Area under the curve; NPV, Negative predictive value; NSTEMI, non ST segment elevation myocardial infarction; PPV, Positive predictive value; Se, sensitivity; Sp, specificity; STEMI, ST segment elevation myocardial infarction; and TTS, Takotsubo syndrome.

## Supplementary Materials

The following are available online at https://www.mdpi.com/2077-0383/9/9/2985/s1, Table S1. Cardiac biomarkers; Table S2. Baseline characteristics of Takotsubo and ACS patients after propensity score; Table S3. In hospital outcome and follow up after propensity score; Table S4. Cardiac biomarkers after propensity score; Table S5. Univariate and multivariate analyses to identify independent predictors of 30-day-mortality; Table S6. Univariate and multivariate analyses to identify independent predictors of 1-year–mortality; Figure S1. TTS vs. STEMI: ROC curves for BNP/TnI at admission (Figure 1a) and at peak (Figure 1b); Figure S2. TTS vs. NSTEMI: ROC curves for BNP/TnI at admission (Figure 2a) and at peak (Figure 2b); Figure S3. TTS vs. STEMI, ROC curve for the complex score; Figure S4. TTS vs. NSTEMI, ROC curve for the complex score; and Figure S5. Proposed new diagnostic algorithm to discriminate TTS from ACS.
