# Supplementary material for: Value of Cardiac Biomarkers in the Early Diagnosis of Takotsubo Syndrome
Source: J Clin Med. 2020 Sep 15;9(9):2985. doi: 10.3390/jcm9092985 (PMC7564647; doi:10.3390/jcm9092985)
Supplement: Supplementary file 1 [file jcm-09-02985-s001.pdf]

SUPPLEMENTARY FILES

Table S1. Cardiac biomarkers .

	TTS patients (*n* = 314)	STEMI patients (*n* = 452)	NSTEMI patients (*n* = 334)	*p* value	*p* value STEMI vs TTS	*p* value NSTEM I vs TTS	*p* value STEMI vs NSTEMI
**Biology at admission**							
Tn I, ng/L	1 [2.7]	1.3 [12]	0.4 [1.5]	<0.001	<0.001	1	<0.001
BNP, ng/mL	427.5 [967.2]	70 [183.2]	99.5 [199.5]	<0.001	<0.001	<0.001	0.445
BNP/Tn	468.2 [1435.5]	26.5 [145.8]	144 [811.0]	<0.001	<0.001	<0.001	0.082
**Biology at peak**							
Tn I, ng/L	2.2 [4.9]	64.3 [138.6]	3.3 [12.1]	<0.001	<0.001	1	<0.001
Day of peak Tn	0 [1]	1 [1]	1 [1]	<0.001	0.484	0.004	<0.001
BNP, ng/mL	666.0 [1203.2]	91 [257]	111 [216.2]	<0.001	<0.001	<0.001	1
Day of peak BNP	0 [2]	0 [0]	0 [0]	0.014	0.100	0.012	0.543
Peak BNP/peak Tn	228.3 [700.1]	1.2 [4.8]	21.4 [107.4]	<0.001	<0.001	<0.001	0.669
**Biology at discharge**							
Troponin I, ng/L	0.4 [1.2]	14.3 [30.8]	1.8 [5.1]	<0.001	<0.001	0.302	<0.001
BNP, ng/mL	323 [501.0]	89 [233]	102 [187]	<0.001	<0.001	<0.001	1

Data presented were number (percentage); quantitative variables as median [interquartile interval] or mean ± 95% confidence interval. BNP, brain natriuretic peptide; NSTEMI, non ST segment elevation myocardial infarction; STEMI, ST segment elevation myocardial infarction; TTS, Takotsubo syndrome; Tn I, Troponin I.

Table S2. Baseline characteristics of Takotsubo and ACS patients after propensity score.

	**TTS patients (*n* = 152)**	**STEMI patients (*n* = 385)**	**NSTEMI patients (*n* = 245)**	***p* value**
**Demographics**				
Age	68.8 ± 13.6	62.8 ± 13.8	67 ± 13.7	<0.001
Female gender, *n* (%)	123 (80.9)	89 (23.1)	58 (23.7)	<0.001
Weight	66.4 ± 15.1	78.7 ± 16.3	81.2 ± 15.8	<0.001
**CVD risk factors**				
Hypertension, *n* (%)	83 (54.6)	197 (51.2)	170 (69.4)	<0.001
Diabetes mellitus, *n* (%)	25 (16.4)	77 (20)	72 (29.4)	0.0035
Dyslipidemia, *n* (%)	53 (34.9)	156 (40.5)	140 (57.1)	<0.001
Current tobacco use, *n* (%)	33 (21.7)	176 (45.7)	76 (31.0)	<0.001
Past tobacco use, *n* (%)	32 (21.1)	101 (26.2)	76 (31.0)	0.088
**Backgroung**				
History of malignancy, *n* (%)	38 (25)	24 (6.2)	15 (6.1)	<0.001
History of psychiatric disease, *n* (%)	44 (29.1)	9 (2.3)	2 (0.8)	<0.001
History of AF, *n* (%)	15 (9.9)	19 (5.0)	25 (10.2)	0.025
History of stroke, *n* (%)	10 (6.6)	23 (6.0)	30 (12.2)	0.014
History of PAD or ischemic heart disease, *n* (%)	29 (19.2)	66 (17.1)	75 (30.6)	<0.001
Chronic kidney disease, *n* (%)				
moderate (DFG 30–60)	30 (19.7)	40 (10.4)	29 (11.8)	
severe (15–30)	3 (2.0)	10 (2.6)	6 (2.4)	-
terminal (<15)	1 (0.7)	2 (0.5)	1 (0.4)	
**Electrocardiogram at admission**				
Q wave, *n* (%)	83 (55.3)	85 (47.5)	4 (1.7)	<0.001
**Echocardiography**				
LVEF at admission, %	39.2 ± 11.9	50.5 ± 11.2	54.5 ± 10.7	<0.001
RV involvement, *n* (%)	4 (2.9)	38 (9.9)	7 (2.9)	<0.001
**Biology at admission**				
CRP, mg/L	7.1 [33.1]	3.0 [8.6]	3.0 [6.9]	<0.001
Leukocytes, G/L	12.6 ± 5.9	11.9 ± 3.9	10.0 ± 5.1	<0.001
Hemoglobin, g/dL	13.3 ± 2.0	14.2 ± 1.7	13.9 ± 1.8	<0.001
Creatinine serum µmol/L	66.0 [30.4]	74.0 [25.6]	75.0 [26.2]	0.0417
GFR, mL/min/m^2	77.7 ± 29.6	81.1 ± 17.2	79.3 ±17.6	0.175
Plasma sodium level, mmol/L	137.7 ± 4.8	137.5 ± 3.1	138.3 ± 2.9	0.00657
Plasma potassium level, mmol/L	4.0 ± 0.5	4.0 ± 0.5	4.0 ± 0.5	0.354

Data presented were number (percentage); quantitative variables as median [interquartile interval] or mean ± 95% confidence interval. AF, atrial fibrillation; CKD-EPI, Chronic Kidney Disease Epidemiology Collaboration; CRP, C-reactive protein; CVD, cardiovascular disease; DBP, diastolic blood pressure; GFR, glomerular filtration rate; LVEF, left ventricular ejection fraction; NSTEMI, non ST segment elevation myocardial infarction; PAD, peripheral artery disease; RV, right ventricle; SBP, systolic blood pressure; STEMI, ST segment elevation myocardial infarction; TTS, Takotsubo syndrome.

Table S3. In hospital outcome and follow up after propensity score.

	**TTS patients (*n* = 152)**	**STEMI patients (*n* = 385)**	**NSTEMI patients (*n* = 245)**	***p* value**
**In-hospital events**				
Cardiogenic shock, *n* (%)	24 (15.8)	11 (2.9)	0 (0)	<0.001
SVT, *n* (%)	34 (22.4)	11 (2.9)	7 (2.9)	<0.001
Sustained VT, *n* (%)	2 (1.3)	1 (0.3)	1 (0.4)	0.2918
3-degree AV block, *n* (%)	1 (0.7)	7 (1.8)	0 (0)	0.07667
In hospital mortality, *n* (%)	12 (7.9)	9 (2.3)	5 (2.0)	0.002173
**Hospital length of stay**, *n* (days)	7.0 [6.0]	5.0 [5.0]	4.0 [4.0]	0.607
**Follow up events**				
Hospitalization for acute HF, *n* (%)	10 (6.8)	22 (6.0)	6 (2.6)	0.1036
30-day mortality, *n* (%)	10 (6.9)	12 (3.1)	7 (2.9)	0.08561
1-year mortality, *n* (%)	20 (15.5)	19 (5.0)	16 (6.6)	<0.001
1-year CV mortality, *n* (%)	7 (4.9)	11 (2.9)	7 (2.9)	0.4627

Data presented were number (percentage); quantitative variables as median [interquartile interval] or mean ± 95% confidence interval; AV, atrio-ventricular; CV, Cardiovascular; HF, heart failure; NSTEMI, non ST segment elevation myocardial infarction; STEMI, ST segment elevation myocardial infarction; SVT, supraventricular tachycardia; TTS, Takotsubo syndrome; VF, ventricular fibrillation; VT, ventricular tachycardia.

Table S4. Cardiac biomarkers after propensity score.

	**TTS patients (*n* = 152)**	**STEMI patients (*n* = 385)**	**NSTEMI patients (*n* = 245)**	***p* value**
**Biology at admission**				
TnI. ng/L	1.7 [3.8]	1.3 [9.9]	0.5 [1.8]	<0.001
BNP, ng/mL	188.0 [432.2]	74.0 [183]	88.0 [152.0]	<0.001
BNP/TnI	136.9 [421.2]	30.0 [144.0]	106.0 [298.0]	0.0398
**Biology at peak**				
TnI, ng/L	4.4 [7.7]	64.2 [129.2]	5.1 [15.4]	<0.001
Day of peak TnI	0 [1.0]	1.0 [1.0]	1.0 [1.0]	<0.001
BNP, ng/mL	411.5 [785.8]	91.0 [236.0]	90.0 [180.0]	<0.001
Day of peak BNP	1.0 [2.0]	0.0 [0.0]	0.0 [0.0]	0.00516
Peak BNP/peak TnI	101.0 [152.3]	1.6 [5.4]	18.1 [59.9]	<0.001
**Biology at discharge**				
TnI, ng/L	0.6 [1.8]	14.3 [30.5]	2.8 [6.7]	<0.001
BNP, ng/mL	252.0 [476.5]	90.0 [224.2]	88.0 [162,0]	<0.001

Data presented were number (percentage); quantitative variables as median [interquartile interval] or mean ± 95% confidence interval. BNP, brain natriuretic peptide; STEMI, ST segment elevation myocardial infarction; TTS, Takotsubo syndrome; Tn I, Troponin I.

Table S5. Univariate and multivariate analyses to identify independent predictors of 30-day- mortality.

	**Univariate Analysis**		**Multivariate Analysis**	
	**OR (95% CI)**	***p* value**	**OR (95% CI)**	***p* value**
**Patient characteristics**				
Age, years	1.03 (1.00–1.07)	0.10		
Female gender	0.64 (0.22–1.85)	0.41		
Hypertension	0.75 (0.30–1.86)	0.53		
Diabetes mellitus	1.32 (0.46–3.79)	0.60		
Dyslipidemia	0.25 (0.07–0.86)	0.03	0.10 (0.01–0.84)	0.03
Current tobacco use	1.45 (0.50–4.17)	0.49		
Past tobacco use	0.63 (0.18–2.21)	0.47		
History of malignancy	2.43 (0.97–6.11)	0.06		
History of psychiatric disease	0.54 (0.17–1.65)	0.28		
History of AF	0.99 (0.28–3.53)	0.99		
History of PAD or ischemic heart disease	2.37 (0.93–6.06)	0.07		
Heart rate, bpm	1.02 (1.00–1.04)	0.06		
SBP, mmHg	0.92 (0.88–0.97)	<0.001	0.92 (0.88–0.96)	<0.001
LVEF on admission, %	0.98 (0.95–1.02)	0.43		
RV involvement	1.18 (0.15–9.59)	0.88		
**Biology on admission**				
Troponin I, ng/L	0.99 (0.91–1.08)	0.88		
BNP, ng/L	1.00 (1.00–1.00)	0.19		
CRP, mg/L	1.01 (1.00–1.01)	0.03	-*	-*
Leukocytes, G/L	1.05 (0.98–1.12)	0.16		
Hemoglobin, g/dl	0.79 (0.63–0.99)	0.04	-*	-*
Creatinine serum, µmol/L	1.00 (1.00–1.00)	0.43		
eGFR, mL/min/1.73 m^2	1.00 (0.98–1.02)	0.86		
Plasma sodium level, mmol/L	0.92 (0.84–1.01)	0.07		

* excluded according to removal criteria. AF, atrial fibrillation; BNP, Brain Natriuretic Peptide; CRP, C-reactive protein; GFR, glomerular filtration rate; LVEF, left ventricular ejection fraction; PAD, peripheral artery disease; RV, right ventricle; SBP, systolic blood pressure.

Table S6. Univariate and multivariate analyses to identify independent predictors of 1-year – mortality.

	Univariate Analysis		Multivariate Analysis	
	**OR (95% CI)**	***p* value**	**OR (95% CI)**	***p* value**
**Patient characteristics**				
Age, years	1.01 (0.99–1.04)	0.21		
Female gender	0.64 (0.30–1.38)	0.25		
Hypertension	0.85 (0.44–1.65)	0.64		
Diabetes mellitus	1.61 (0.76–3.39)	0.22		
Dyslipidemia	0.27 (0.12–0.62)	0.002	0.18 (0.07–0.47)	<0.001
Current tobacco use	1.01 (0.43–2.34)	0.99		
Past tobacco use	1.30 (0.61–2.79)	0.49		
History of malignancy	3.66 (1.85–7.22)	<0.001	-*	-*
History of psychiatric disease	0.83 (0.40–1.71)	0.61		
History of AF	0.68 (0.25–1.87)	0.46		
History of PAD or ischemic heart disease	1.55 (0.76–3.17)	0.22		
Heart rate, bpm	1.02 (1.01–1.04)	0.002	1.02 (1.00–1.03)	0.04
SBP, mmHg	0.95 (0.92–0.98)	0.003	-*	-*
LVEF on admission, %	0.95 (0.93–0.98)	0.004	0.95 (0.92–0.98)	0.004
RV involvement	6.50 (1.97–21.48)	0.002	-*	-*
**Biology on admission**				
Troponin I, ng/L	1.01 (0.97–1.06)	0.56		
BNP, ng/L	1.00 (1.00–1.00)	0.008	-*	-*
CRP, mg/L	1.01 (1.00–1.01)	<0.001	-*	-*
Leukocytes, G/L	1.06 (1.01–1.11)	0.02	-*	-*
Hemoglobin, g/dl	0.73 (0.62–0.87)	<0.001	0.76 (0.63–0.93)	0.006
Creatinine serum, µmol/L	1.00 (1.00–1.01)	0.25		
eGFR, mL/min/1.73 m^2	0.99 (0.97–1.00)	0.047	-*	-*
Plasma sodium level, mmol/L	0.90 (0.84–0.97)	0.004	-*	-*

* excluded according to removal criteria. AF, atrial fibrillation; BNP, Brain Natriuretic Peptide; CRP, C-reactive protein; GFR, glomerular filtration rate; LVEF, left ventricular ejection fraction; PAD, peripheral artery disease; RV, right ventricle; SBP, systolic blood pressure.

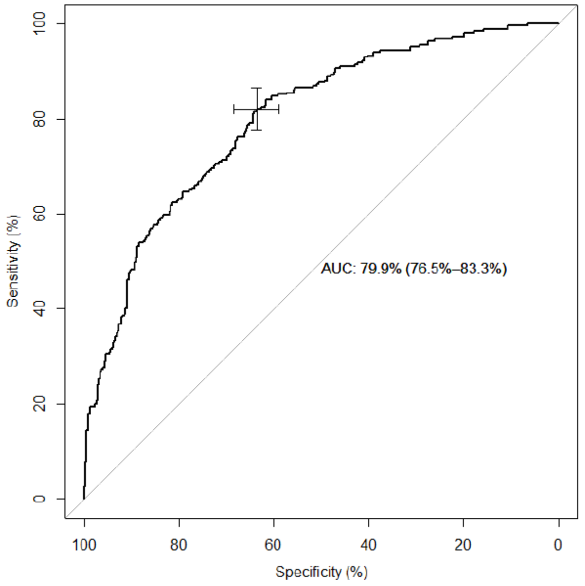

STEMI 68.25 TAKO TSUBO

Figure a. TTS *vs* STEMI, ROC curve for BNP/TnI at admission (supplementary files)

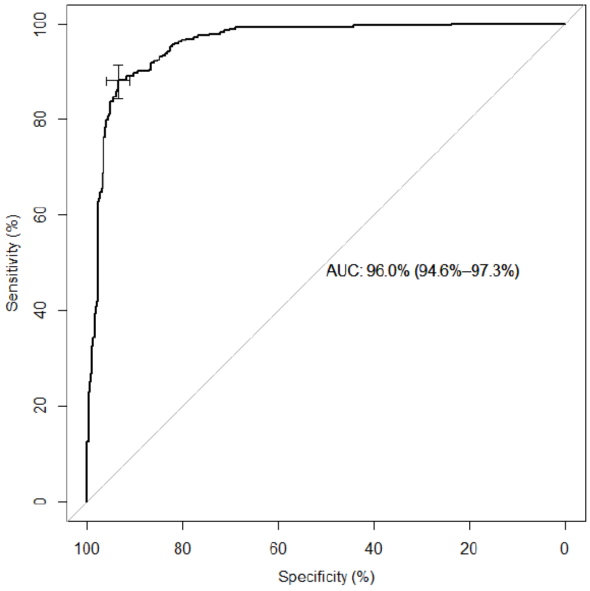

STEMI 39.25 TAKO TSUBO

Figure b. TTS *vs* STEMI, ROC curve for BNP/TnI at peak (supplementary files)

Figure S1. TTS *vs* STEMI: ROC curves for BNP/TnI at admission (Figure a) and at peak (Figure b); BNP, B-natriuretic peptide; STEMI, ST segment elevation myocardial infarction; Tn, Troponin; TTS, Takotsubo syndrome

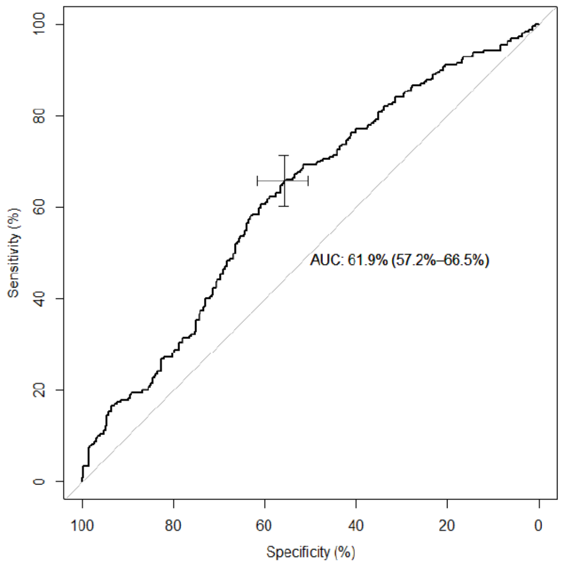

NSTEMI	196.25	TAKO TSUBO

Figure a. TTS *vs* NSTEMI, ROC curve for BNP/TnI at admission (supplementary files)

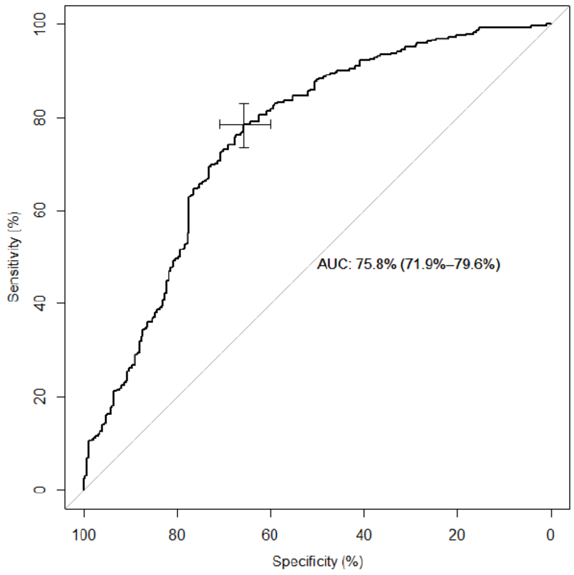

NSTEMI	76.50	TAKO TSUBO

Figure b. TTS *vs* NSTEMI, ROC curve for BNP/TnI at peak (supplementary files)

Figure S2. TTS *vs* NSTEMI: ROC curves for BNP/TnI at admission (Figure a) and at peak (Figure b); BNP, B-natriuretic peptide; NSTEMI, non ST segment elevation infarction; Tn, Troponin; TTS, Takotsubo syndrome.

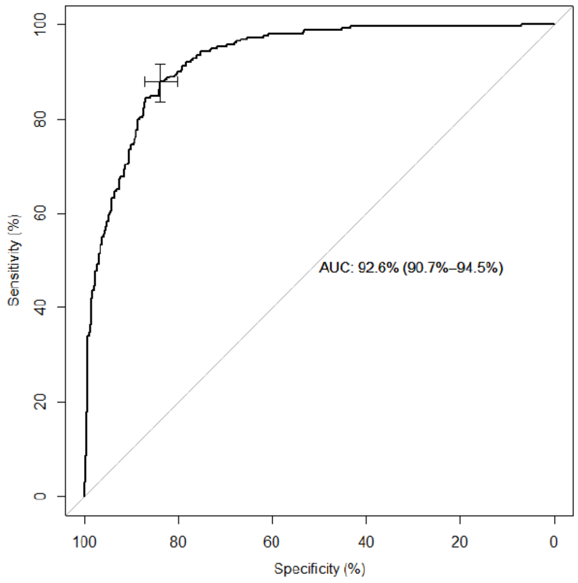

STEMI	0.3851	TAKO TSUBO

Figure S3. TTS *vs* STEMI, ROC curve for the complex score; STEMI, ST segment elevation myocardial infarction; TTS, Takotsubo syndrome

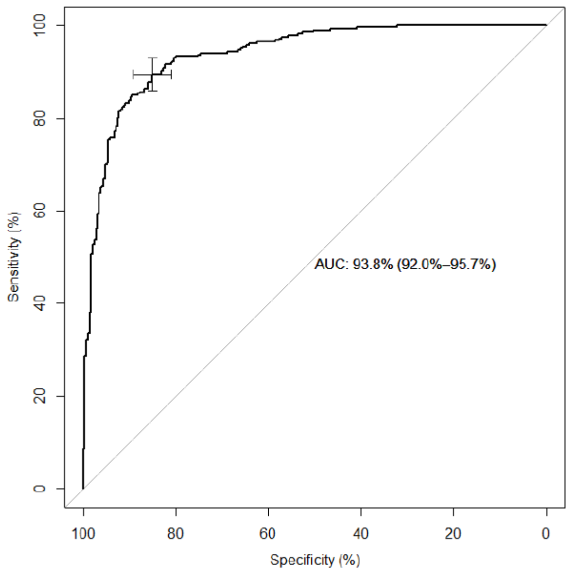

NSTEMI 0.4406 TAKO TSUBO

Figure S4. TTS *vs* NSTEMI, ROC curve for the complex score; NSTEMI, non ST segment elevation infarction; TTS, Takotsubo syndrome.

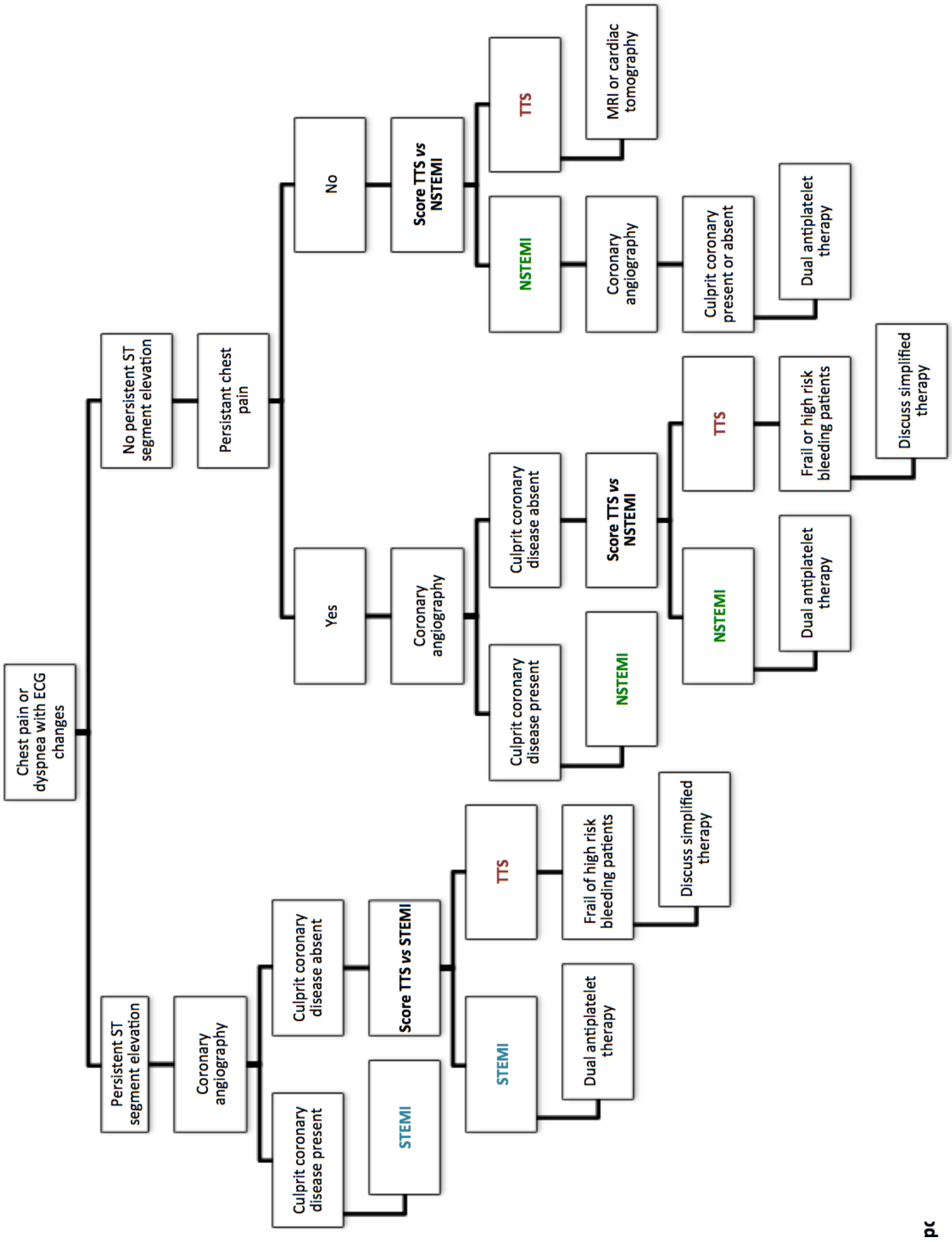

Figure S5. Proposed new diagnostic algorithm to discriminate TTS from ACS; NSTEMI, non ST segment elevation infarction; STEMI, ST segment elevation myocardial infarction; TTS, Takotsubo syndrome.
